# Improving working memory by electrical stimulation and cross-frequency coupling

**DOI:** 10.1186/s13041-024-01142-1

**Published:** 2024-10-01

**Authors:** Wiam Al Qasem, Mohammed Abubaker, Kateřina Pilátová, Petr Ježdík, Eugen Kvašňák

**Affiliations:** 1 Department of Medical Biophysics and Medical Informatics, Third Faculty of Medicine, Charles University in Prague, Prague, Czechia; 2 Department of Information and Communication Technology in Medicine, Faculty of Biomedical Engineering, Czech Technical University in Prague, Prague, Czechia; 3 Department of Circuit Theory, Faculty of Electrical Engineering, Czech Technical University in Prague, Prague, Czechia

**Keywords:** Working memory (WM), Transcranial alternating current stimulation (tACS), Theta-gamma peak-coupled transcranial alternating current stimulation, Power spectral density (PSD), Electroencephalography (EEG)

## Abstract

Working memory (WM) is essential for the temporary storage and processing of information required for complex cognitive tasks and relies on neuronal theta and gamma oscillations. Given the limited capacity of WM, researchers have investigated various methods to improve it, including transcranial alternating current stimulation (tACS), which modulates brain activity at specific frequencies. One particularly promising approach is theta-gamma peak-coupled-tACS (TGCp-tACS), which simulates the natural interaction between theta and gamma oscillations that occurs during cognitive control in the brain. The aim of this study was to improve WM in healthy young adults with TGCp-tACS, focusing on both behavioral and neurophysiological outcomes. Thirty-one participants completed five WM tasks under both sham and verum stimulation conditions. Electroencephalography (EEG) recordings before and after stimulation showed that TGCp-tACS increased power spectral density (PSD) in the high-gamma region at the stimulation site, while PSD decreased in the theta and delta regions throughout the cortex. From a behavioral perspective, although no significant changes were observed in most tasks, there was a significant improvement in accuracy in the 14-item Sternberg task, indicating an improvement in phonological WM. In conclusion, TGCp-tACS has the potential to promote and improve the phonological component of WM. To fully realize the cognitive benefits, further research is needed to refine the stimulation parameters and account for individual differences, such as baseline cognitive status and hormonal factors.

**Supplementary Information:**

The online version contains supplementary material available at 10.1186/s13041-024-01142-1.

## Introduction

### Working memory

Working memory (WM) is crucial for the temporary storage and processing of information and supports higher cognitive abilities such as logical thinking, problem solving and the understanding of complex concepts [1–3]. Baddeley's model of WM is widely used in cognitive psychology and is divided into three interrelated components [3]: the visuospatial sketchpad, which handles visual and spatial information [4, 5]; the phonological loop, which processes verbal and auditory data [6]; and the central executive, which orchestrates these components and performs key functions such as updating WM representations (updating function), transitioning between task rules (switching function), and inhibiting irrelevant responses (inhibition function) [7]. WM has a limited capacity and its impairments are associated with neurological and psychiatric disorders such as schizophrenia, mild cognitive impairment (MCI), attention-deficit/hyperactivity disorder (ADHD), and Alzheimer's disease (AD), indicating the critical role of WM in mental health [8–12]. Various cognitive tests have been developed to assess WM functionality, each tailored to evaluate different components and functions, such as the visuospatial sketchpad and inhibitory function [13–15]. Even in abbreviated form, these tasks have been shown to be effective for measuring various WM components and have provided valuable insights into the structure of our cognitive processes [16].

### Brain oscillations and brain stimulation

Brain oscillations or neuronal oscillations are the rhythmic electrical activity generated by neuronal tissue in response to stimuli [17]. These oscillations occur in different frequency bands—delta, theta, alpha, beta and gamma—and are involved in several functional processes in the brain [18, 19]. All neuronal oscillations are involved in WM processing, especially the theta and gamma frequencies [20]. The interaction between neuronal oscillations is referred to as cross-frequency coupling (CFC), which can manifest itself in various forms [21]. One of the best-known forms of coupling associated with WM processing is theta/gamma phase-amplitude coupling (PAC), in which the amplitude of gamma oscillations is modulated by the phase of theta waves. This phenomenon is often referred to as the theta/gamma neural code [22–24]. The theta/gamma PAC is thought to support the representation and maintenance of multiple WM elements. Two models have been proposed: one assumes that each gamma wave within a theta cycle represents a single memory item, with WM capacity possibly limited by the number of gamma waves that can fit into a theta cycle [25, 26]. The second model assumes that an entire gamma burst within a theta cycle encodes a single memory item [27, 28].

Brain oscillations can be modulated by various methods, e.g. sensory entrainment [29, 30], non-invasive brain stimulation (NIBS) [31, 32] and invasive techniques [33, 34]. Among the NIBS methods, transcranial electrical stimulation (tES) and transcranial magnetic stimulation (TMS) are widely used in both research and clinical settings [35, 36]. tES, which includes techniques such as transcranial direct current stimulation (tDCS) and transcranial alternating current stimulation (tACS), is particularly favored for its accessibility, tolerability, and cost-effectiveness [37, 38]. tDCS affects neuronal activity by either increasing excitability through anodal stimulation or decreasing excitability through cathodal stimulation [39–42]. In contrast, tACS modulates brain function by using fluctuating currents to synchronize cerebral networks at specific frequencies—a capability that tDCS does not offer [43, 44]. The prevailing view is that tACS directly influences neuronal networks in the cortex during stimulation [45, 46], with its aftereffects—such as increased oscillatory activity post-stimulation—likely resulting from synaptic changes promoted by spike-timing-dependent plasticity (STDP) [47, 48]. Given the link between irregular cortical oscillations, CFC and various neuropsychiatric and neurodegenerative disorders [44, 49–51], tACS holds promise for treating brain diseases and improving cognitive function through frequency- and phase-specific modulation of cortical oscillations [52, 53]. However, to achieve optimal stimulation, various parameters such as location, intensity, frequency and dosage need to be carefully considered. As there are no standardized protocols yet, these factors need to be carefully adjusted to achieve effective results.

### Transcranial-alternating current stimulation and working memory

Numerous research studies have investigated the effects of tACS on WM [54–57], with a particular focus on theta and gamma frequencies, which have attracted considerable interest due to their potential to improve WM performance [58–64]. However, the results are inconsistent, likely due to differences in study methods, such as differences in stimulation parameters, target areas, intensity, and participant characteristics [54, 56, 60–72]. This variability underscores the need for more standardized research. Individual factors also appear to influence the efficacy of tACS in improving WM. Evidence suggests that tACS may be particularly effective for individuals with lower baseline performance and when applied during more cognitively demanding tasks [66, 68, 70, 71, 73–76]. Among the various tACS techniques investigated, theta/gamma peak-coupled-tACS (TGCp-tACS) has shown particular promise. In this innovative approach, gamma bursts are synchronized with the peaks of theta waves to improve WM [77]. Initial studies, especially applied to the left frontal cortex, have yielded encouraging results [77]. In particular, TGCp-tACS, which delivers gamma bursts in the range of 80–100 Hz synchronized with the peaks of theta waves oscillating around 6 Hz, has been associated with significant improvements in visuospatial WM [77]. In another study, the potential of TGCp-tACS was confirmed by demonstrating improved performance in the modified Sternberg task when stimulation occurred at tuned frequencies [78]. Despite these positive results, further research is needed to confirm the efficacy of TGCp-tACS, optimize stimulation protocols, and investigate its broader application in different cognitive tasks and populations.

Building on the limited yet promising research regarding TGCp-tACS and its impact on WM, this study aimed to evaluate the effects of this non-invasive stimulation technique applied to the left frontal cortex. Both the behavioral effects, in particular the stimulation-induced changes in performance across five different WM tasks, and the neurophysiological effects, reflected in TGCp-tACS-induced alterations in the electroencephalography (EEG) power spectrum, in healthy young adults were investigated. To increase detection sensitivity, we used a comprehensive set of five different WM tasks, each designed to assess different aspects of WM function. An overview of the tasks used to assess WM components in this study is provided in Additional File 1, which also outlines the methods and frameworks frequently employed in related research [94–116]. Detailed descriptions of the tasks used in this study can be found in the “Materials and Methods” section.

## Materials and methods

The study was approved by the Ethics Committee of the Third Faculty of Medicine of Charles University in Prague and complied with the principles of the Declaration of Helsinki. Exclusion criteria included metal implants in the head, implanted electronic devices, seizures, mental or neurological disorders, strokes, substance abuse, history of neurological problems or head trauma, use of psychotropic drugs, use of drugs that alter neuronal activity, and left-handedness. Before participating in the study, the volunteers provided written informed consent. This study involved 31 right-handed, non-color-blind medical students (16 females, ages 19.8 ± 1.61) with normal or corrected-to-normal vision. None of the participants had any contraindications to tACS, and all were naïve to both the tasks and the stimulation.

### Experimental procedure

**Fig. 1:**
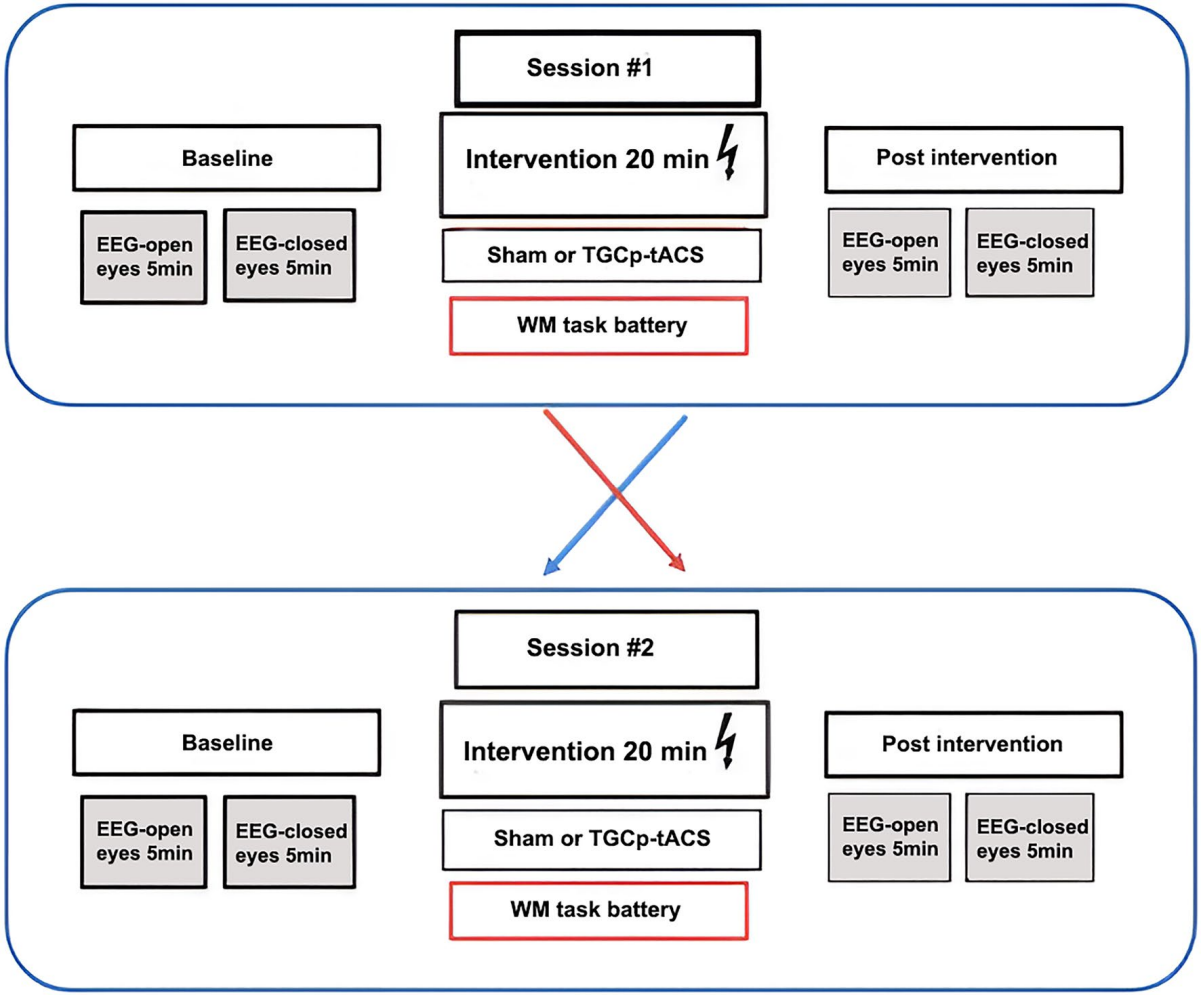
Experimental setup of the study. Session # 1 and session # 2 are separated by at least 72 h. TGCp-tACS: theta/gamma peak coupled transcranial-alteranating current stimulation; WM:working memory; EEG: electroencephalography

Two sessions (sham and verum stimulation) were performed at least 72 h apart. The order of the sessions was counterbalanced between the participants. All subjects attended a face-to-face introductory session to familiarize them with the laboratory and the procedure. Each session consisted of 1) a 5-min pre-stimulation EEG recording in the resting state (eyes-open), followed by 2) a 5-min resting state EEG recording (eyes-closed), 3) a 20-min sham or verum stimulation during which participants completed the WM task battery, 4) a 5-min post-stimulation EEG recording in the resting state (eyes-open), followed by 5) a 5-min resting state EEG recording (eyes-closed). Figure 1 illustrates the experimental setup.

### EEG recording and stimulation setup

All experiments were performed in a laboratory free of sound and electromagnetic signals. The Starstim® wireless hybrid tES-EEG neurostimulator system with NIC v2.0.11.7 software (Neuroelectrics Ltd., Barcelona, Spain) was used for electrical stimulation and EEG recording. EEG was recorded with Ag/AgCl electrodes placed at 20 standard positions according to the international 10–20 system. EEG activity was recorded at 20 scalp locations (Fp1, Fp2, Fz, F3, F4, F7, F8, Cz, C3, C4, T7, T8, Pz, P3, P4, P7, P8, Oz, O1 and O2). Electrical stimulation was provided by five NG-Pistim electrodes (1 cm radius and π cm^2^ contact area) filled with conductive EEG gel (a next-generation hybrid electrode that can be used for EEG monitoring and stimulation). The central electrode was placed over F3, and four return electrodes were equally spaced around the central electrode (Fp1, Fz, C3, F7) so that high focal stimulation of the left frontal cortex was achieved. The stimulation signal had a sampling rate of 1 MHz and an analogue-to-digital precision of 14 bits (≈0.5 μA), and the electrode impedance was kept below 10 kOhm.

### Theta/gamma peak coupled-tACS protocol

An alternating current of 1 mA peak to baseline was applied for 20 min (including 10 s fade-in and 10 s fade-out time). Stimulation consisted of two overlapping components: a continuous slow theta wave at 6 Hz (0.6 mA peak to baseline) and gamma bursts at 80 Hz (0.4 mA peak to baseline). Gamma bursts lasted 50 ms during each peak and were synchronized with the continuous theta wave. The temporally accurate fusing of the components was achieved by dedicated hardware and monitored with an oscilloscope. tACS was applied for 20 min while participants performed the WM battery test. The procedure and stimulation characteristics (current intensity and frequency) in the sham condition were identical to those in the verum condition, except for the stimulation duration, which was only 30 s and then automatically switched off.

### WM test battery

The test subjects were seated 50 cm in front of a PC with a 24" monitor (resolution 2560 × 1440, 60 Hz) and the WM tests were carried out using the E-prime® software. Before carrying out the actual experiment, they completed a number of standard exercises for each task. The order of the tasks was counterbalanced and pseudorandomized between subjects during the actual experiment. The WM test battery comprised the following five subtests [Visuospatial WM task, Sternberg task, Digit Symbol Substitution Test (DSST), Flanker task and Wisconsin Card Sorting Test (WCST)], which correspond to the specific components of WM; the description of each task can be found in Table 1.

**Table 1:** Description of working memory task battery

WM task	WM component	Task description
Visuospatial WM task	Visuospatial sketchpad	In the Visuospatial WM task, participants were presented with a sequence of red and blue rectangles displayed at different angles for 500 ms. The aim is to memorize the angles of the red rectangles located on a particular side of the screen, which is indicated by an arrow. After a retention time of 900 ms, participants were shown a test screen that could correspond to the memory screen. If the test screen exactly matched the memory screen, participants were asked to press “1” on the keyboard; if they did not match, they were instructed to press “2”. On each trial, two or four red rectangles (stimuli) appeared continuously, accompanied by 2, 3, 4, 5 or 6 distracting blue rectangles. Each participant went through nine cycles with a total of 72 trials
Sternberg WM task	Phonological loop	In the Sternberg task, participants were first presented with a mix of green and black letters and were asked to memorize only the black ones, ignoring the green ones. Each letter was displayed for 1000 ms. Following this, they were shown a series of red letters, each displayed for 3000 ms, and the participants had to respond within this time frame. Their task was to decide if a specific red letter matched any of the black letters they had memorized. They pressed “1” on the keyboard if it matched and “2” if it didn't. This task was conducted in four cycles, each with two parts. In the first part, participants memorized eight letters and then responded to 14 test letters (14-item condition), In the second part, they memorized a different set of eight letters and responded to 10 test letters (10-item condition), which is less cognitively demanding
Digit Symbol Substitution Test (DSST)	Central executive components and processing speed	In the DSST Test, participants needed to quickly match numbers to their corresponding symbols using a provided key. When symbols appeared on the screen, they had to identify the correct numbers accurately and swiftly. They had 180 s to create as many correct digit-symbol pairs as possible within this time
Flanker Task	Central executive: Inhibition	In the flanker task, participants had to determine the direction of the middle arrow in a row of arrows. They pressed “1” on the keyboard when the arrow pointed to the left and “2” when it pointed to the right. The task comprised 141 trials, each displayed for 1000 ms, and fell into one of three categories: congruent, neutral, or incongruent. The participates had to respond within a 1 min timeframe for each trial. In the congruent condition, the center arrow matched the direction of the surrounding arrows (e.g., < < < < <). In the incongruent condition, the middle arrow pointed in the opposite direction of the surrounding arrows (e.g., < < > < <), creating a conflict. In the neutral condition, the middle arrow was flanked by neutral symbols, providing a different challenge (e.g., )
Wisconsin Card Sorting Test (WCST)	Central executive: Switching	In the WCST, participants are asked to categorize cards according to various criteria, such as color, number or shape of symbols. Over the course of 42 trials, each lasting 5000 ms, the participants sort the cards according to dynamically changing criteria. The categorization rule is changed after every 14 cards, so that the participants have to adapt their sorting strategy flexibly. After each sorting attempt, the participants receive feedback informing them of the correctness or incorrectness of their performance

WM: working memory

### EEG preprocessing

The EEG data were preprocessed using a customized MATLAB script based on the EEGLab Toolbox [79]. First, the raw EEG data were imported into EEGLab and the positions of 20 electrodes were identified, including FP1, FP2, F7, F3, Fz, F4, T7, C3, C4, T8, P7, P3, P4, P8, O1, Oz and O2. The EEG data were then resampled to 256 Hz and the direct current (DC) offset was eliminated before filtering the data. A high-pass finite impulse response (FIR) filter with a cut-off frequency of 0.5 Hz was then applied. Artifact rejection of the EEG data was performed using the EEGLAB plugin clean_rawdata, where channels were flagged as bad if they had a prolonged flatness of more than 5 s, showed significant noise values (defined as a low signal-to-noise ratio with a standard deviation of more than 4), or had poor correlation with neighboring channels. Artifact subspace reconstruction (ASR) algorithms were then used to remove corrupted data segments. The raw data time series were visually inspected to remove any additional artifacts, followed by the application of average referencing. To remove artifacts due to eye movements and muscle activity, an independent component analysis (ICA) algorithm was run, and then the IClabel plugin was used to identify and remove artifactual components. All removed channels were then interpolated to maintain data integrity. Data preprocessing was performed according to the instructions in the EEGLAB tutorials and established pipelines.

### Power spectrum analysis

The time series data was initially transformed into the frequency domain using the fast Fourier transform (FFT) based on the Welch method. A Hamming window of 256 points with a 50% overlap (128 points) was applied. The power spectra obtained from these windows were averaged and then converted to a logarithmic scale. The mean power spectral density (PSD) for the delta (δ: 1–4 Hz), theta (θ: 4–7 Hz), alpha (α: 8–12 Hz), beta (β: 13–30 Hz), gamma (γ: 30–70 Hz), and high gamma (γ: 70–100 Hz) bands across 20 channels was then plotted on a two-dimensional (2D) topographic map.

### Statistical analysis

Generalized Linear Mixed-Effects Model (GLMM) fitted by Penalized Likelihood (PL) were used to analyze the behavioral data, accounting for the complex data structure, including repeated measures on the same participants in different conditions. In addition, a paired t-test was used to determine PSD differences between groups. The null hypothesis was rejected at probability values below 0.05. To minimize type I error, the false discovery rate (FDR) method was used for multiple comparisons. Statistical PSD analysis was performed using FieldTrip implemented in the EEGLAB environment [79, 80].

## Results

### Behavioral results

#### Visuospatial WM

In the Visuospatial WM task, the statistical analysis primarily focused on accuracy and reaction time (RT) under two conditions: 2 stimuli (2 red rectangles) and 4 stimuli (4 red rectangles). RT is the interval between the presentation of a stimulus and the participant's response to it. The analysis revealed that TGCp-tACS did not have a significant effect on either measure. For the 2-stimuli condition, TGCp-tACS resulted in a non-significant change in accuracy by 1.92% (p = 0.22, 95% CI − 1.18 to 5.02%) and a non-significant increase in RT by 5.06 ms (p = 0.59, 95% CI − 13.44 to 23.58 ms). For the 4-stimuli condition, TGCp-tACS led to a non-significant change in accuracy by 0.91% (p = 0.64, 95% CI − 2.90 to 4.72%) and a non-significant increase in RT by 17.42 ms (p = 0.07, 95% CI − 1.70 to 36.54 ms). These results indicate that TGCp-tACS does not significantly impact accuracy or RT in the Visuospatial WM task among young participants under both 2-stimuli and 4-stimuli conditions.

#### DSST

In the DSST analysis for young participants, TGCp-tACS did not result in a significant change in accuracy or RT. Specifically, TGCp-tACS showed a negligible increase in accuracy of 0.07% (p = 0.72, 95% CI − 0.31 to 0.45%). In terms of RT, TGCp-tACS resulted in a non-significant decrease in RT of 18.18 ms (p = 0.07, 95% CI − 37.86 ms to 1.50 ms). These results indicate that TGCp-tACS has no significant effect on accuracy or RT in DSST in young participants.

#### Flanker task

In the Flanker task conducted with young participants, the statistical analysis primarily focused on RT because of the very low or nonexistent error rate. This lack of variability in the data makes accuracy an unreliable measure for analysis. TGCp-tACS did not have a significant effect on RT across neutral, congruent, and incongruent conditions. For the neutral condition, TGCp-tACS resulted in a non-significant decrease in RT by 3.69 ms (p = 0.19, 95% CI − 9.15 ms to 1.76 ms). In the congruent condition, TGCp-tACS led to a negligible change in RT by − 0.36 ms (p = 0.90, 95% CI − 5.76 ms to 5.05 ms). For the incongruent condition, the change in RT was 0.81 ms (p = 0.82, 95% CI − 6.19 ms to 7.82 ms) due to TGS-tACS. Table 2 shows the changes in RTs due to TGCp-tACS in different conditions of the Flanker task.

**Table 2:** Reaction time changes due to theta/gamma peak-coupled transcranial alternating current stimulation in different conditions of the Flanker task

Condition	Change in RT (ms) due to TGCp-tACS	p-value	95% Confidence Interval
Neutral	− 3.69	0.19	[− 9.15, 1.76]
Congruent < < < < <	− 0.36	0.90	[− 5.76, 5.05]
Incongruent < < > < <	0.81	0.82	[− 6.19, 7.82]

RT: reaction time; TGCp-tACS: theta/gamma peak-coupled transcranial-alternating current stimulation

#### WCST

In the WCST, the statistical analysis focused primarily on the number of perseverative errors and the RT. The analysis revealed that the TGCp-tACS had no significant effect on either measure. For the number of perseverative errors, TGCp-tACS resulted in a non-significant change of − 0.01 (p = 0.5, 95% CI − 0.03 to 0.02). In terms of RT, TGCp-tACS resulted in a non-significant reduction of 23.79 ms (p = 0.20, 95% CI − 59.76 ms to 12.37 ms). These results show that TGCp-tACS has no significant effect on the number of perseverative errors or the RT in WCST in young participants.

#### Sternberg task

**Fig. 2:**
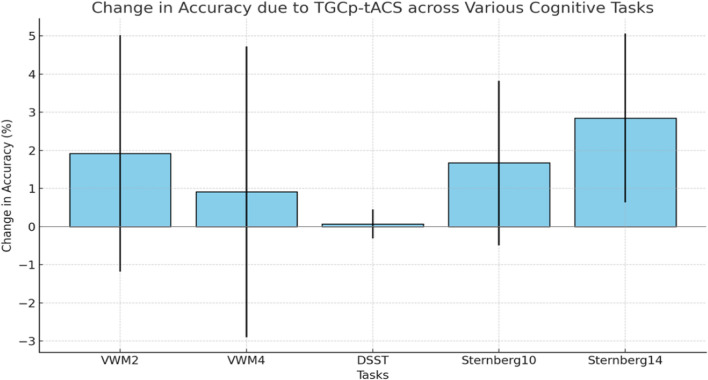
Effect of TGCp-tACS on accuracy across various cognitive tasks. This bar graph illustrates the change in accuracy due to TGCp-tACS across various cognitive tasks. The error bars represent the 95% confidence intervals for each task condition. The change in accuracy was only statistically significant for the 14-item Sternberg task. TGCp-tACS: Theta/gamma peak coupled-transcranial-alternating current stimulation; VWM2: visuospatial working memory (2-stimulus); VWM4: visuospatial working memory (4-stimulus); DSST: Digit Symbol Substitution Task; Sternberg10: Sternberg task (10-item); Sternberg14: Sternberg task (14-item)

In the Sternberg task conducted with young participants, the statistical analysis revealed that TGCp-tACS did not have a significant effect on either accuracy or RT in the 10-item condition, with a change in accuracy of 1.67% (p = 0.13, 95% CI − 0.49 to 3.82%) and a change in RT of 25.20 ms (p = 0.15, 95% CI − 9.03 to 59.43 ms). In the 14-item condition, TGCp-tACS significantly improved accuracy by 2.84% (p = 0.01, 95% CI 0.63 to 5.06%). However, TGCp-tACS did not significantly affect RT in the 14-item condition, with a change of − 18.61 ms (p = 0.22, 95% CI − 48.18 to 10.96 ms). These results indicate a significant improvement in accuracy in the 14-item condition but no significant impact on RT in either condition. Figure 2 illustrates the graphical representation of the accuracy changes in the different task conditions used in this study.

### Electrophyisological results

#### Sham condition

**Fig. 3:**
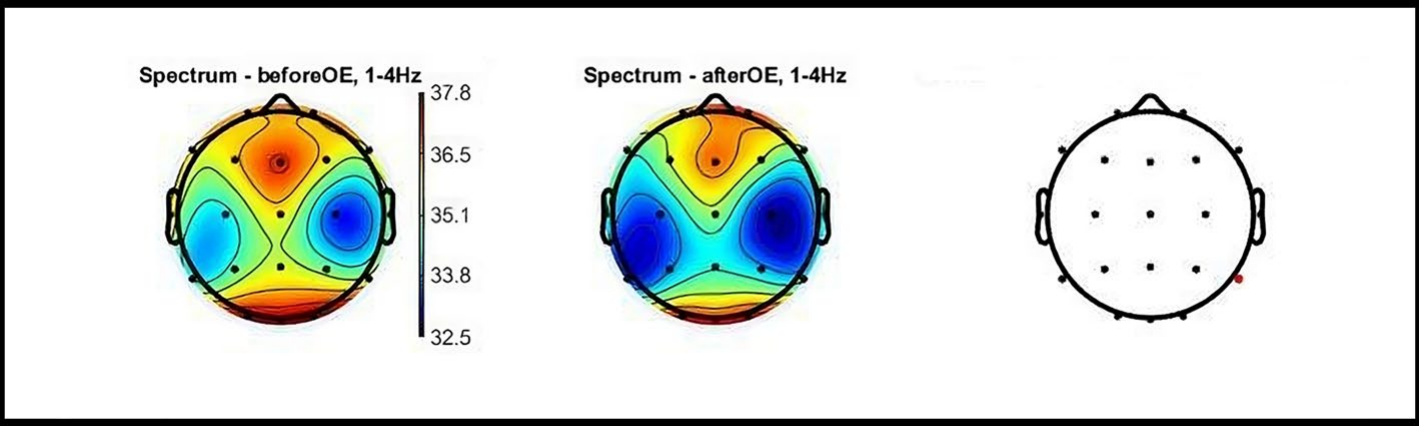
Topographic Maps of PSD Distribution in Delta Range: Sham Condition OE. Topographic 2D maps depicting PSD distribution across different electrodes in the delta range. These maps highlight statistically significant differences observed before and after the sham condition with eyes open (red dots). Statistical analysis was conducted using paired t-tests with false FDR correction for multiple comparisons (p < 0.05) utilizing the Fieldtrip toolbox. OE: eyes open; CE: eyes closed; Hz: hertz; PSD: Power spectral density; FDR: false discovery rate

- Eyes-open (immediately after completion of the task and sham stimulation) compared to eyes-open (before the task and sham stimulation).
  - General decrease in delta and theta power in all brain regions.
  - Statistically significant decrease in delta power at electrode P8 in the right parietal region. Figure 3 shows topographic 2D maps depicting PSD distribution across different electrodes in delta range, highlighting the statistically significant differences observed before and after sham condition with eyes-open.
  - Minimal increase in beta and gamma PSD values.
- Eyes-closed (at least 5 min after completion of the task and sham stimulation) compared to eyes-closed (before the task and sham stimulation).
  - Minor fluctuations in PSD values in all frequency bands.
  - Brain activity tended to return to the baseline values observed before the task.

#### Verum condition

**Fig. 4:**
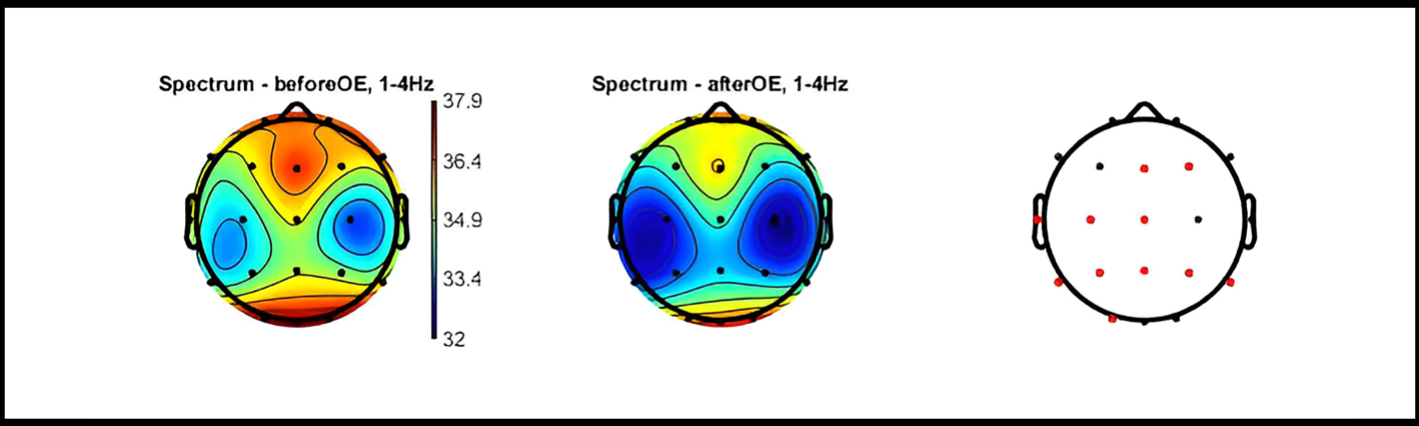
Topographic Maps of PSD Distribution in Delta Range: Verum Condition OE. Topographic 2D maps depicting PSD distribution across different electrodes in the delta range. These maps highlight statistically significant differences observed before and after the verum condition with eyes open (red dots). Statistical analysis was conducted using paired t-tests with FDR correction for multiple comparisons (p < 0.05) utilizing the Fieldtrip toolbox. OE: eyes open; CE: eyes closed; Hz: hertz; PSD: Power spectral density; FDR: false discovery rate

**Fig. 5:**
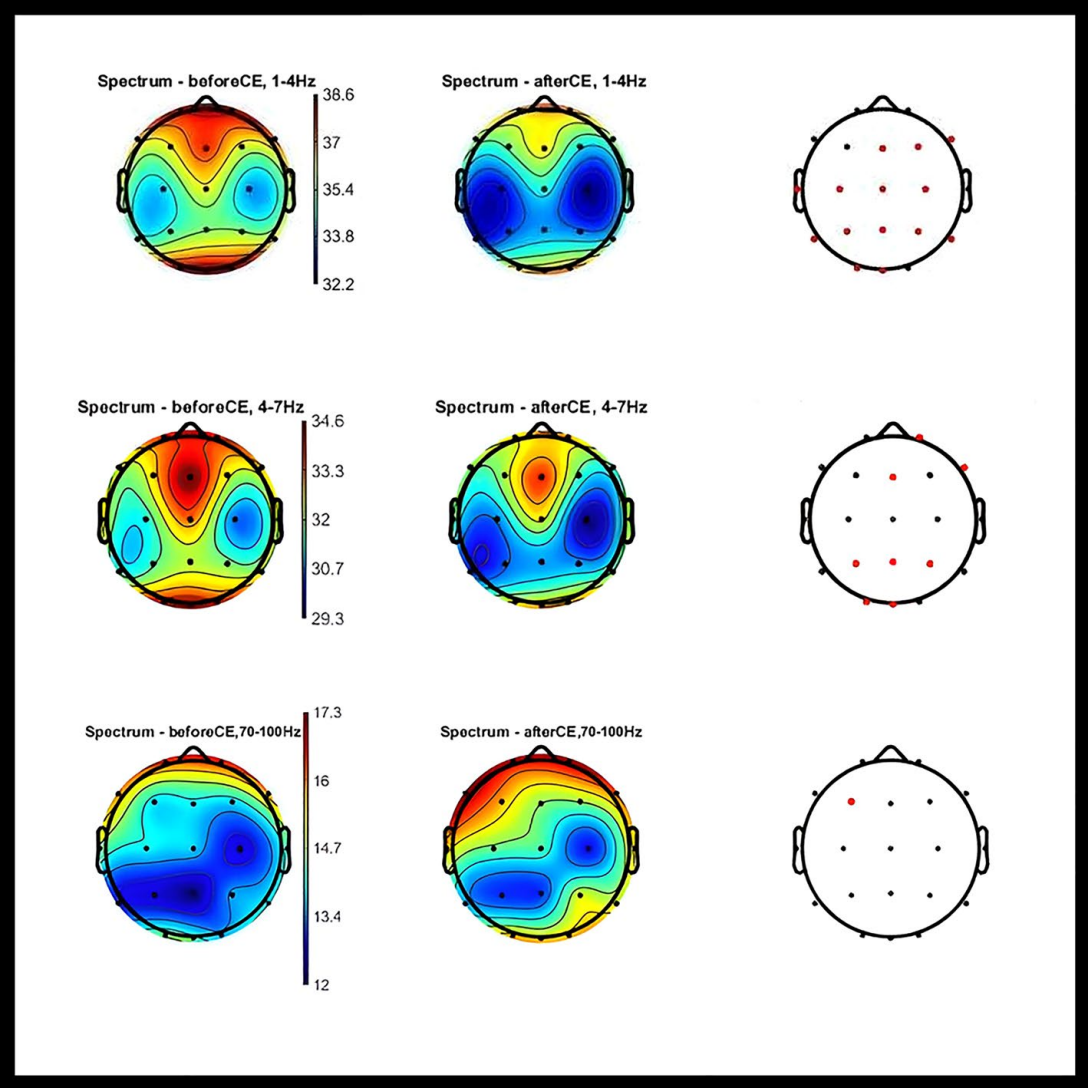
Topographic Maps of PSD Distribution in Delta, theta and high-gamma Ranges: Verum Condition CE. These maps highlight statistically significant differences observed before and after the verum condition with eyes closed (red dots). Statistical analysis was conducted using paired t-tests with false discovery rate (FDR) correction for multiple comparisons (p < 0.05) utilizing the Fieldtrip toolbox. OE: eyes open; CE: eyes closed; Hz: hertz; PSD: Power spectral density; FDR: false discovery rate

- Eyes-open (immediately after completion of the task and verum stimulation) compared to eyes-open (before the task and verum stimulation).
  - Statistically significant global decrease in delta PSD. For more details refer to Fig. 4
  - Non-significant global decrease in theta PSD.
  - Negligible changes in PSD at the other frequencies.
- Eyes-closed (at least 5 min after completion of the task and verum stimulation) compared to eyes-closed (before the task and verum stimulation).
  - Statistically significant decrease in delta and theta power in a large cortical area.
  - Statistically significant increase in PSD in the high gamma range (70–100 Hz) at the site of stimulation (F3). For more details refer to Fig. 5.

## Discussion

### Behavioral outcomes

This study examined the effects of a single TGCp-tACS session on WM in young, healthy participants, focusing on both behavioral and neurophysiological outcomes. To increase sensitivity in detecting stimulation effects, five different WM tasks were used, each targeting a specific WM component (e.g., visuospatial sketchpad, phonological loop, executive functions). Specifically, TGCp-tACS was found to significantly improve accuracy only on the 14-item Sternberg task, which assesses the phonological component of WM. Previous studies have primarily examined the effects of TGCp-tACS on the visuospatial sketchpad and phonological components of WM. Our results are in contrast to those of [77], who found significant improvements in visuospatial WM using a visuospatial match-to-sample test with TGCp-tACS at a theta frequency of 6 Hz and gamma bursts between 80 and 100 Hz delivered to the left frontal cortex. In contrast to their results, we found only a slight and non-significant improvement in visuospatial WM. Furthermore, our study protocol differed somewhat from that of [78], who administered frequency-tuned TGCp-tACS to the left frontal cortex and reported improvements in a modified Sternberg task. Although we did not use frequency-tuned TGCp-tACS, we found a significant improvement in the accuracy of the Sternberg task, but only at high cognitive demands. This suggests that despite the methodological differences, there is a remarkable parallel in the results. Several factors could explain the behavioral outcomes of TGCp-tACS in our study: The chosen intensity and frequencies of tACS (6 Hz and 80 Hz) may not have been optimally matched to the natural frequencies required for effective neuromodulation [81]. The participants, high-performing medical students with an average age of 19.8 years, may have been close to their maximum potential for WM capacity and cognitive performance, leading to ceiling effects. It is also possible that most of the tasks were not challenging enough to show a benefit of TGCp-tACS in these high-performing individuals.

Research has shown that hormonal fluctuations during the menstrual cycle can significantly affect cognitive function, including memory, attention, and executive functions [82, 83]. Furthermore, [84] found that cortical excitability in women, or the degree to which the cerebral cortex responds to stimuli, aligns with that of men only during the follicular phase of the menstrual cycle. In our study, which included 16 female participants, there was a minimum 72-h interval between the experimental and sham sessions, during which we did not specifically investigate the hormonal effects on WM outcomes. Therefore, it is possible that participants were in different phases of their menstrual cycle during each session, which could potentially influence the interpretation of the TGCp-tACS effects on WM.

### Neurophysiological findings

Verum stimulation led to a significant increase in gamma power at the F3 position and a decrease in delta and theta power in several cortical regions. These effects were most pronounced in the eyes-closed EEG condition, which was recorded at least five minutes after stimulation and task completion. This suggests that the external sensory input in the eyes-open condition and the task-related dynamic changes measured immediately after completion of stimulation may mask the actual effects of stimulation. In the post-sham eyes-open EEG recordings, there was a notable reduction in the PSD of the delta and theta frequency bands and minimal increase in the beta and gamma PSD, suggesting a shift to a more alert and focused state shortly after task completion. In contrast, with sham eyes-closed condition, brain activity largely returned to baseline at least five minutes following the end of the task completion, with only minor fluctuations in PSD in all frequency bands. This comparison suggests that the effects observed in the eyes-closed verum condition are due to the stimulation itself. The reduced sensory input in the eyes-closed condition leads to a more pronounced resting state, which improves the detectability of the TGCp-tACS-induced changes. As mentioned above, the persistent brain activity after tACS indicates lasting changes in synaptic plasticity rather than mere entrainment per se. By administering alternating currents at specific frequencies, tACS can synchronize brain rhythms, which may affect the timing of presynaptic and postsynaptic spikes, thereby increasing or decreasing synaptic strength through STDP, leading to long-term potentiation (LTP) and long-term depression (LTD), respectively [85]. It has been suggested that administration of tACS at frequencies at or slightly below the endogenous frequency leads to LTP, while higher frequencies lead to LTD. In addition, STDP is associated with power changes in EEG frequency bands after stimulation [86]. In our study, the administered theta frequency of 6 Hz may have been significantly different from the endogenous frequencies of the participants, leading to LTD and a decrease in theta PSD. Conversely, the administered gamma frequency of 80 Hz may have been closer to the endogenous gamma frequency, which likely led to LTP and an increase in gamma PSD. Furthermore, tACS could induce frequency-unspecific modulation of neuronal oscillations [58], as indicated by decrease in delta PSD. The decrease in theta activity caused by LTD could indirectly diminish delta oscillations due to the strong coupling and interaction between these frequencies [87, 88].

### Limitations and future directions

The study is limited by several factors: suboptimal tACS frequencies, potential ceiling effects due to high-performing participants, insufficiently challenging WM tasks, uncontrolled hormonal fluctuations, reliance on a single tACS session, and the specific characteristics of the participants limit the generalizability of the findings to a broader population.

To better determine the effects of TGCp-tACS on WM, several strategies can be used:

1. Recruit participants from different educational and occupational backgrounds to show how TGCp-tACS affects different cognitive levels and to attenuate potential ceiling effects.
2. Ensure that the cognitive tasks are sufficiently challenging for high achieving young adults.
3. Stimulation parameters such as frequency and intensity should be tailored to the unique neurophysiological characteristics of each individual, and the administration of multiple stimulation sessions (e.g., 5 sessions/ week for several weeks) could enhance the stimulation effects [89, 90].
4. Consider hormonal influences on WM in females by conducting verum and sham sessions in the same menstrual phase, ideally 28 days apart to reduce variability [91, 92]. Starting the first session in the follicular phase may align women’s cortical excitability with that of men, enabling a more accurate comparison [93].

## Conclusion

Application of TGCp-tACS to the left frontal cortex of young, healthy adults resulted in a significant improvement in accuracy on the cognitively demanding Sternberg task, which measures phonological WM. However, it had no significant effects on other WM components, indicating the need for further refinement of stimulation methods. Neurophysiological data showed that verum stimulation increased high-gamma PSD at the site of the stimulation and decreased theta and delta PSD throughout the cortex. Although these results are promising, further research is needed to optimize stimulation parameters, with a focus on modulating lower gamma frequencies and individual theta frequencies that can be accurately identified from EEG recordings either before stimulation or within a closed loop, as well as accounting for individual differences, such as baseline cognitive status and hormonal influences, to fully exploit the cognitive enhancement potential of TGCp-tACS.

## Supplementary Information

Additional file 1

## Data Availability

The datasets used and/or analyzed during the current study are available from the corresponding author on reasonable request.

## Abbreviations

2D: Two-dimensional
AD: Alzheimer’s disease
ADHD: Attention-deficit/hyperactivity disorder
ASR: Artifact subspace reconstruction
CE: Eyes-closed
CFC: Cross-frequency coupling
DSST: Digit Symbol Substitution Test
EEG: Electroencephalography
FDR: False discovery rate
FFT: Fast Fourier transform
GLMM: Generalized Linear Mixed-Effects Model
ICA: Independent component analysis
LTP: Long-term potentiation
LTD: Long-term depression
MCI: Mild cognitive impairment
NIBS: Non-invasive brain stimulation
OE: Eyes-open
PAC: Phase-amplitude coupling
PL: Penalized Likelihood
PSD: Power spectral density
RT: Reaction time
STDP: Spike-timing-dependent plasticity
TGCp-tACS: Theta/gamma peak-coupled tACS
tACS: Transcranial alternating current stimulation
tDCS: Transcranial direct current stimulation
tES: Transcranial electrical stimulation
WCST: Wisconsin Card Sorting Test
WM: Working memory
