# Supplementary material for: Improving working memory by electrical stimulation and cross-frequency coupling
Source: Mol Brain. 2024 Oct 1;17:72. doi: 10.1186/s13041-024-01142-1 (PMC11446076; doi:10.1186/s13041-024-01142-1)
Supplement: Supplementary file 1 — Additional file 1 [file 13041_2024_1142_MOESM1_ESM.docx]

**Additional file 1** Overview of Tasks Utilized to Assess Working Memory Components.

| **Wisconsin card sorting task (WCST)** |
| --- |
| - **Assessment of the cognitive domain: Executive function (switching)** [94,95] - **Key measured variables:**  Perseverative errors, RT - **Description and findings:**   - Participants have to categorize the cards according to certain criteria (color, shape, number), then the rule for categorizing the cards changes after a certain number of cards and participants have to adapt to the changed rule and adhere to the new rule (details in Methods section)   - Available in different forms (e.g., a comprehensive version with 128 cards, the WCST-64 variant and the WCST-3 variant, which ends after completing the first three categories) [96,97].   - A certain number of correct answers or cards may be required to change the rule for categorizing the cards [97–100].   - An important indicator of cognitive flexibility is the number of perseverative errors, which indicate the inability of participants to change their response tactics in response to rule changes [95,100,101]. |
| **Digit symbol substitution task (DSST)** |
| - **Assessment of the cognitive domain**: **Executive functions and processing speed** [102] - **Key measured variables:**  Accuracy, RT - **Description and findings:**   - Participants are first presented with a key containing digits paired with certain symbols and are then asked to match the following symbols to the corresponding digit as quickly and accurately as possible (details in the method section) [103].   - Versions of the task: paper-and-pencil version and computerized versions [102,104,105]   - The DSST reflects processing speed and a broader range of cognitive and executive abilities, such as inhibition, cognitive flexibility and updating [7,106,107] |
| **Sternberg task** |
| - **Assessment of the cognitive domain**: Phonological loop - **Key measured variables:**  accuracy, RT - **Description and findings:**   This task involves three sequential phases: Encoding, Retention and Retrieval.   - - In the encoding phase, participants are briefly presented with verbal/visual information (usually letters) [108–111]   - In the retention phase, the verbal information is memorized through subvocal practice,   - In the retrieval phase, the stored information is used to formulate a response   - The visual stimuli are converted into a phonological format for storage, and the verbal stimuli are stored directly without the need for conversion [1,112]. |
| **Flanker task** |
| - **Assessment of the cognitive domain**: Executive function (inhibition) - **Key measured variables:**  accuracy, RT - **Description and findings:**   - People are asked to quickly and accurately recognize the direction in which the central arrow is pointing while ignoring the surrounding distractions [113–115].   - These distractions can be congruent, incongruent or neutral with respect to the direction of the central arrow. |
| **Working memory task** |
| - **Assessment of the cognitive domain**: visuospatial sketchpad - **Key measured variables:**  accuracy, RT - **Description and findings:**   - During this task, participants view an array of colored squares displayed on a screen. Following a short delay, a second array appears, and participants need to identify whether it matches the first array. Some task variations include distractors, such as differently colored rectangles, to evaluate the participant's capacity to filter out irrelevant information (see Methods section for details) [116]. |

WCST: Wisconsin card sorting task; RT: reaction time; DSST: Digit symbol substitution task.
